# Real-world bleeding rates on emicizumab: the value of using nationwide digital treatment diary data in clinical research

**DOI:** 10.1016/j.rpth.2025.102717

**Published:** 2025-02-27

**Authors:** Martijn R. Brands, Elisabeth M. Taal, Martijn Oude Voshaar, Mariëtte H.E. Driessens, Caroline M.E. van Veen, Marieke J.H.A. Kruip, Paul L. den Exter, Britta A.P. Laros-van Gorkom, Marjet A. Stein-Wit, Kathelijn Fischer, Stephan Meijer, Karina Meijer, Marlène Beijlevelt, Karin Fijnvandraat, Samantha C. Gouw

**Affiliations:** 1 Department of Pediatric Hematology, Amsterdam UMC location University of Amsterdam, Amsterdam, the Netherlands; 2 Department of Clinical Epidemiology, Leiden University Medical Center, Leiden, the Netherlands; 3 HemoNED Foundation, Leiden, the Netherlands; 4 Department of Public Health, Erasmus University Medical Center Rotterdam, Rotterdam, the Netherlands; 5 Netherlands Hemophilia Patient Society (NVHP), Nijkerk, the Netherlands; 6 Department of Hematology, Erasmus MC, Erasmus University Medical Center Rotterdam, Rotterdam, the Netherlands; 7 Department of Medicine, Thrombosis and Hemostasis, Leiden University Medical Center, Leiden, the Netherlands; 8 Department of Hematology, Radboud University Medical Center, Nijmegen, the Netherlands; 9 Hemophilia Treatment Center Nijmegen-Eindhoven-Maastricht, Nijmegen, the Netherlands; 10 Department of Pediatrics, University Medical Center Groningen, Groningen, the Netherlands; 11 Center for Benign Haematology, Thrombosis and Haemostasis, Van Creveldkliniek, University Medical Center Utrecht, Utrecht University, Utrecht, the Netherlands; 12 PedNet Haemophilia Research Foundation, Baarn, the Netherlands; 13 Department of Hematology, University Medical Center Groningen, University of Groningen, Groningen, the Netherlands; 14 Department of Molecular Cellular Hemostasis, Sanquin Research and Landsteiner Laboratory, Amsterdam, Netherlands, the Netherlands

**Keywords:** emicizumab, hemophilia A, patient-generated health data, registries, telemedicine

## Abstract

**Background:**

People with hemophilia in the Netherlands log bleeds and infusions through a digital treatment diary. With the current innovations in hemophilia treatments, the use of patient-reported bleeding data will become increasingly important.

**Objective:**

To assess real-world bleeding rates on emicizumab in a nationwide cohort of people with severe hemophilia A, and assess the value of digital treatment diary data.

**Methods:**

People with severe hemophilia A of all ages with and without inhibitors using emicizumab who use the digital treatment diary were included. From 2018 to October 2023, data on bleeds treated with clotting factor concentrate were collected from digital treatment diaries and electronic health records. Mean (95% CI) annualized (joint) bleeding rates were calculated using negative-binomial regression analyses. Proportions of people with zero-treated (joint) bleeds were assessed using Kaplan–Meier survival analysis. We calculated the proportion of all bleeds that were recorded in digital treatment diaries.

**Results:**

The 232 included persons (median age, 27 years; IQR, 13-51) who used emicizumab for a median of 27 months (IQR, 14-31 months). The mean treated annualized bleeding rate and annualized joint bleeding rate were 1.5 (CI, 1.3-1.8) and 0.8 (CI, 0.6-1.0), respectively. At 24 weeks, 63% had zero-treated bleeds, and 80% had zero-treated joint bleeds. Of treated bleeds, 67% (310/460) were reported in digital treatment diaries.

**Conclusion:**

Bleeding rates among Dutch people with severe hemophilia A using emicizumab were comparable to other real-world studies. We formulated recommendations to improve the quality of patient-reported bleeding data, such as establishing guidelines for recording bleeds and improving interoperability.

## Essentials

• There is limited real-world data on bleeds among people with hemophilia using emicizumab.
• Treated bleeds reported in digital treatment diaries and electronic health records were assessed.
• Negative-binomial regression model-based (joint) bleeding rates were comparable with other studies.
• Of all identified treated bleeds, 67% were reported by individuals in digital treatment diaries.

## 1 Introduction

Hemophilia A is caused by a deficiency of coagulation factor (F)VIII (FVIII). Based on the residual FVIII activity, its severity is classified as severe (FVIII activity <0.01 IU/mL), moderate (0.01-0.05 IU/mL), or mild (0.06-0.40 IU/mL) [1]. People with severe hemophilia A experience spontaneous joint and muscle bleeds, resulting in joint damage. Before 2020, most people with severe hemophilia A used intravenous FVIII concentrate therapy as prophylaxis, usually administered 2 to 3 times weekly. Since 2020, a significant proportion of Dutch people with severe hemophilia A switched to emicizumab for prophylaxis [2]. Emicizumab is a bispecific monoclonal antibody that mimics the function of coagulation FVIII and can be administered subcutaneously 1 to 4 times per month. In addition to a reduced treatment burden, emicizumab has been suggested to result in fewer (joint) bleeds compared to conventional FVIII prophylaxis. Clinical trials demonstrated a reduction of 79% among people ≥12 years with inhibitors [3], 99% among children <12 years with inhibitors [4], and 68% among people ≥12 years without inhibitors, compared with previous FVIII prophylaxis [5]. Until now, knowledge of the real-world bleeding rates among people with severe hemophilia A using emicizumab for prophylaxis is limited. Two Dutch studies have previously evaluated emicizumab in the Utrecht cohort, as presented in Supplementary Table S4 [6].

All Dutch persons with hemophilia who infuse treatment at home are asked to log all prophylactic and therapeutic infusions and bleeds in a digital treatment diary [7]. The digital treatment diary was introduced in 2018. Its use has been promoted nationwide. Health care providers can access treatment diary data to evaluate treatment efficacy and shared decision-making on treatment plans. The treatment diary data are migrated to the national Dutch patient registry for persons with hemophilia and other congenital bleeding disorders (HemoNED), to enable secondary use of data [8]. With the current innovations eliciting profound changes in hemophilia treatment, the secondary use of patient-reported (registry) data will become increasingly important to evaluate the long-term effects and safety of therapies [9,10]. Understanding the value and limitations of patient-reported bleed data collected through a digital treatment diary is required to improve implementation, and to allow for robust design of future studies.

In this study, we assessed the real-world (joint) bleeding rates among people with severe hemophilia A using emicizumab prophylaxis in a population-wide cohort in the Netherlands. Second, we aimed to assess the value of using digital treatment diary data to determine bleeding rates.

## 2 Methods

### 2.1 Participants

**Figure 1:**
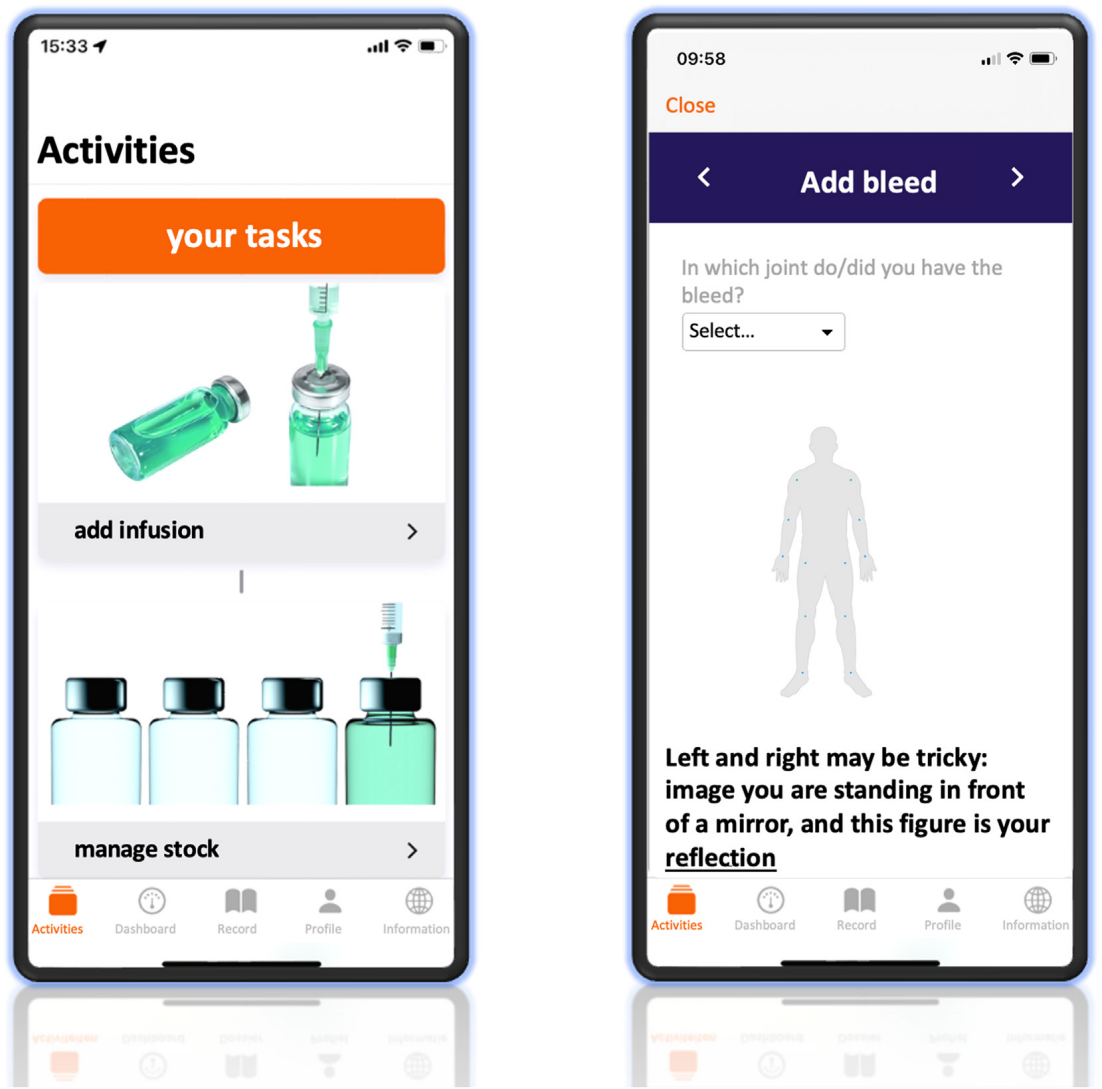
VastePrik treatment diary application [7]. The application is only available in Dutch, and is translated for the purpose of this article.

In this observational cohort study, all children and adults with severe hemophilia A using emicizumab prophylaxis in the Netherlands, regardless of inhibitor status, who have registered bleeds or infusions in the digital treatment diary “VastePrik” (Figure 1) [7] since initiation of emicizumab prophylaxis were eligible for inclusion. Participants who discontinued emicizumab prophylaxis during the follow-up period were included in analyses until their stop date.

### 2.2 Data extraction

All bleeds treated with clotting factor concentrates that occurred after the 4-week loading dose phase of emicizumab were extracted from the digital treatment diary. We included all identified bleeds, including those treated in hospitals, at home, or where the location of treatment was unknown. Bleeding data were complemented using data from electronic health records. Bleeds were cross-referenced based on the date of occurrence. Data obtained between 2018 and October 2023 were analyzed. For each bleed, the following characteristics were extracted: cause (trauma, surgery, activity-induced, spontaneous, and other), type (joint, muscle, mucosal, soft tissue/subcutaneous, intracranial, and gastrointestinal), location, treatment dose(s), and number of days treated.

### 2.3 Outcomes and measures

Annualized bleeding rates (ABRs), annualized joint bleeding rates (AJBRs), and the proportions of people with zero-treated bleeds were assessed. Bleeds were defined as all bleeding events for which coagulation factor concentrate was administered at least once. To evaluate clinically relevant major bleeds, we also assessed the subset of bleeds for which factor concentrate was administered ≥2 days [11]. Bleeding rates for traumatic and nontraumatic bleeds were calculated. Nontraumatic bleeds were defined as bleeds with the following reported cause: “spontaneous,” “activity induced,” and “other.”

Finally, we assessed the value of using digital treatment diary data for the assessment of bleeding rates, by calculating the proportion of all (joint) bleeds that were recorded in the digital treatment diary. We also calculated the proportion of participants who did not report any treated (joint) bleeds in their treatment diary.

### 2.4 Data analysis

Descriptive statistics were used and reported as median values with IQR. Mean (95% CI) bleeding rates were calculated by negative-binomial regression analyses. This is the most optimal method to account for different follow-up times, by including a log-conversion of the follow-up years as an offset variable. Zero bleed rates were assessed at 24 weeks, 1 year, and 2 years by Kaplan–Meier survival analyses. Subgroup analyses were conducted for participants without current inhibitors, pediatric participants, and adult participants. We determined bleeding rates in different age categories, but a formal comparison was not possible due to limited numbers per category.

### 2.5 Ethics

All individuals who are registered in the Dutch Haemophilia Registry provided written consent for the use of their data for research purposes and data checks to increase data quality (Institutional Review Board of the Leiden University Medical Centre, P16.129).

## 3 Results

### 3.1 Participant characteristics

**Figure 2:**
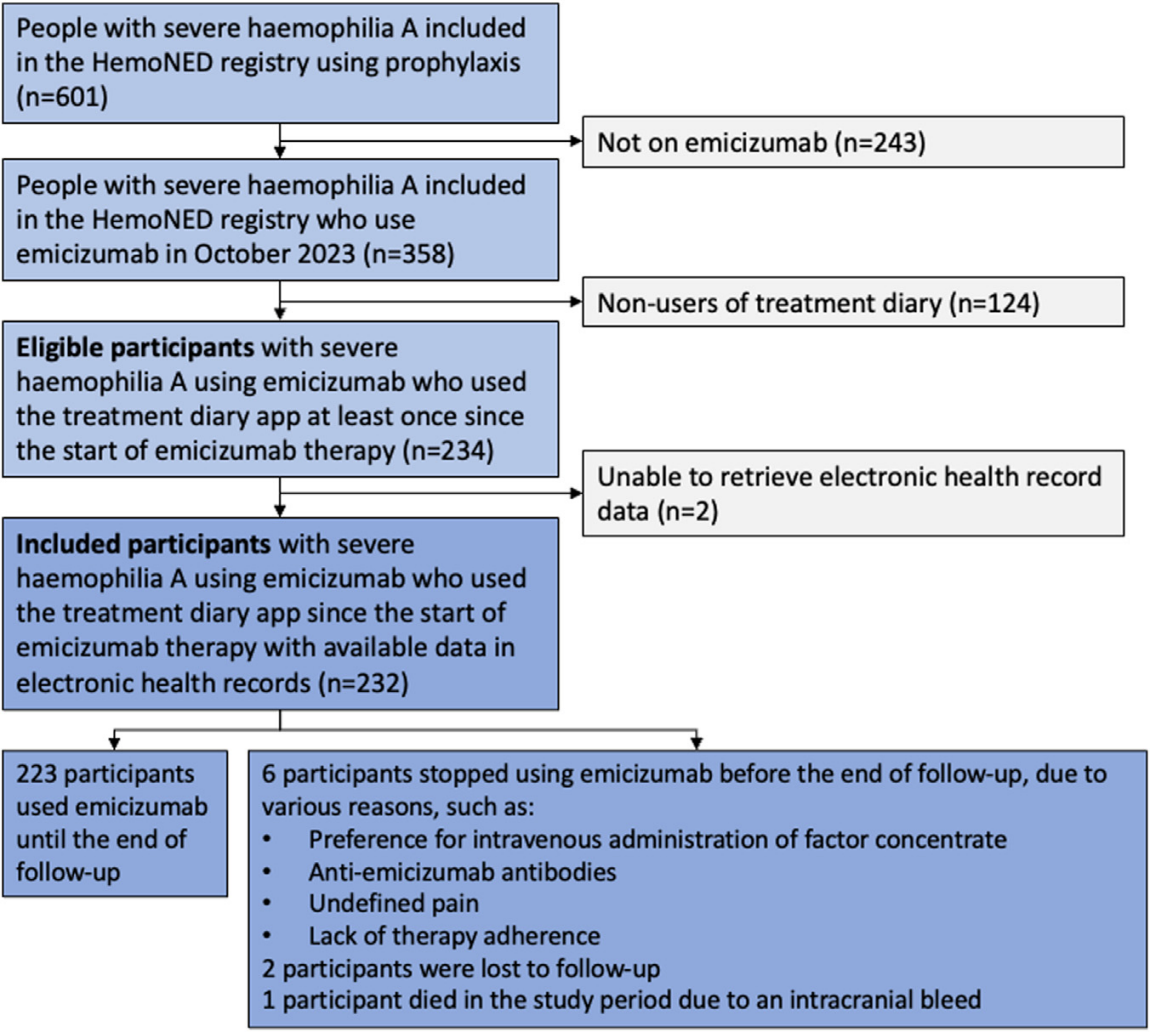
Study flowchart.

In total, 232 participants were included in this study, as illustrated in the study flowchart (Figure 2). The median duration of emicizumab use was 27 months (IQR, 14-31 months), as shown in Table 1. The total number of participants’ treatment years was 469. The median (IQR) emicizumab injection frequency at prescription was once every 2 (1-2) weeks. Their median age at the start of emicizumab was 27 years (IQR, 13-51 years). The majority of participants were adults (63%, 147/232) and 37% (85/232) were children. In 14% (11/85) of children, inhibitors were present at the start of emicizumab prophylaxis. For adults, this was 5% (7/147). During the follow-up period, 14 participants reported side effects at the injection site: redness (11 participants), itching (9), sensitive skin (8), warmth (3), and pain (2). Three participants reported joint pain as a side effect, 1 experienced undefined leg pain, and 1 participant tested positive for antiemicizumab antibodies. Nine participants stopped using emicizumab before the end of the study period for reasons explained in Figure 2.

**Table 1:** Characteristics of included male participants with severe haemophilia A.

Characteristic	All participants (*n* = 232)	Children (*n* = 85)	Adults (*n* = 147)
Age in years at the start of emicizumab, median (IQR)	27 (13-51)	8 (3.5-14.5)	44 (29-59)
On demand treatment at the start of emicizumab, *n* (%)	11 (5%)	11 (15%)	0
HIV infection, *n* (%)	11 (5%)	0	11 (8%)
Hepatitis C infection, *n* (%)			
Current infection	2 (1%)	0	2 (2%)
Treated or cleared	71 (30%)	0	71 (48%)
Never infected	159 (69%)	85 (100%)	74 (50%)
Inhibitor, *n* (%)			
Current inhibitor	18 (8%)	11 (14%)	7 (5%)
Past inhibitor	37 (16%)	12 (14%)	25 (17%)
Never had an inhibitor	177 (76%)	62 (72%)	115 (78%)
Emicizumab prescription (initial)^a^, median (IQR)			
Injection frequency, once every n week(s)	2 (1-2)	2 (2-3)	2 (1-2)
Dose in mg/kg/wk	1.48 (1.40-1.57)	1.50 (1.34-1.65)	1.48 (1.40-1.56)
Missing	52 (22%)	41 (48%)	11 (7.5%)
Follow-up duration in months^b^, median (IQR)	27 (14-31)	29 (22-32)	25 (11-31)

a Dose and frequency of emicizumab prophylaxis after the loading dose phase of 28 days.

b The follow-up period starts 28 days after the start of emicizumab prophylaxis, until the moment of data collection (October 1, 2023) or the stop date of emicizumab prophylaxis.

The 124 excluded persons who did not use the digital treatment diary were slightly younger (median age: 19 years) than our study population, as presented in Supplementary Table S1. Other characteristics were similar.

### 3.2 Bleeding rates on emicizumab

The mean model-based ABR for all participants was 1.5 (95% CI, 1.3-1.8), and the mean ABR for major bleeds treated ≥2 days was 0.8 (95% CI, 0.7-0.9), as presented in Table 2. In children, the ABR was 1.4 (95% CI, 1.1-1.8), and 0.7 (95% CI, 0.5-1.0) for major bleeds treated ≥2 days. In adults, the mean ABR was 1.6 (95% CI, 1.3-1.9), and 0.8 (95% CI, 0.7-1.0) for major bleeds treated ≥2 days. The highest mean ABR is found among participants aged 45 to 60 and ≥60 years, and adolescents aged 10 to 17 years. In a sensitivity analysis restricted to 214 participants without current inhibitors, ABRs were comparable with all 232 participants, as presented in Supplementary Table S2.

**Table 2:** Model-based bleeding rates on emicizumab, per age category.

Bleeding rate	All participants (*n* = 232)	Children			Adults				
		All children (*n* = 85)	0-9 y (*n* = 45)	10-17 y (*n* = 40)	All adults (*n* = 147)	18-30 y (*n* = 46)	31-44 y (*n* = 31)	45-60 y (*n* = 36)	>60 y (*n* = 34)
ABR, mean (95% CI)									
Treated bleeds	1.5 (1.3-1.8)	1.4 (1.1-1.8)	1.0 (0.7-1.3)	1.9 (1.3-2.6)	1.6 (1.3-1.9)	1.2 (0.8-1.7)	1.2 (0.8-1.7)	2.1 (1.5-3.0)	2.0 (1.3-3.1)
Bleeds treated ≥2 d	0.8 (0.7-0.9)	0.7 (0.5-1.0)	0.3 (0.2-0.5)	1.2 (0.8-1.6)	0.8 (0.7-1.0)	0.7 (0.5-1.0)	0.8 (0.5-1.2)	0.9 (0.6-1.4)	1.0 (0.6-1.6)
AJBR, mean (95% CI)									
Treated joint bleeds	0.8 (0.6-1.0)	0.6 (0.5-0.8)	0.4 (0.3-0.7)	0.8 (0.6-1.2)	0.9 (0.7-1.2)	0.5 (0.3-0.8)	0.6 (0.4-1.1)	1.4 (0.9-2.2)	1.1 (0.6-2.1)
Joint bleeds treated ≥2 d	0.4 (0.3-0.5)	0.4 (0.3-0.5)	0.2 (0.1-0.4)	0.6 (0.4-0.8)	0.4 (0.3-0.6)	0.2 (0.1-0.4)	0.4 (0.2-0.8)	0.6 (0.3-1.1)	0.6 (0.3-1.1)
Traumatic AJBR, mean (95% CI)									
Treated joint bleeds	0.4 (0.3-0.5)	0.4 (0.3-0.6)	0.4 (0.2-0.6)	0.4 (0.3-0.7)	0.3 (0.2-0.4)	0.2 (0.1-0.4)	0.3 (0.1-0.5)	0.4 (0.2-1.1)	0.3 (0.2-0.5)
Joint bleeds treated ≥2 d	0.2 (0.1-0.2)	0.2 (0.2-0.3)	0.2 (0.1-0.3)	0.3 (0.2-0.5)	0.1 (0.1-0.2)	0.1 (0.1-0.2)	0.2 (0.1-0.3)	0.1 (0.1-0.3)	0.1 (0.1-0.3)
Nontraumatic AJBR, mean (95% CI)									
Treated joint bleeds	0.4 (0.3-0.6)	0.2 (0.1-0.3)	0.1 (0.0-0.1)	0.4 (0.2-0.6)	0.6 (0.4-0.9)	0.2 (0.1-0.4)	0.3 (0.1-0.8)	1.0 (0.6-1.7)	0.9 (0.4-1.9)
Joint bleeds treated ≥2 d	0.2 (0.2-0.3)	0.2 (0.1-0.2)	0.0 (0.0-0.1)	0.3 (0.2-0.5)	0.3 (0.2-0.5)	0.1 (0.0-0.3)	0.2 (0.1-0.6)	0.5 (0.2-1.0)	0.4 (0.2-1.0)

ABR, annual bleeding rate; AJBR, annual joint bleeding rate.

### 3.3 Joint bleeding rates on emicizumab

The mean AJBR for all participants was 0.8 (95% CI, 0.6-1.0), and for major joint bleeds treated ≥2 days it was 0.4 (95% CI, 0.3-0.5). For nontraumatic joint bleeds and nontraumatic major joint bleeds treated ≥2 days, the mean AJBRs were 0.4 (95% CI, 0.3-0.6), and 0.2 (95% CI, 0.2-0.3). In children, the AJBR was 0.6 (95% CI, 0.5-0.8), and 0.4 (95% CI, 0.3-0.5) for major bleeds treated ≥2 days. In adults, the mean (95% CI) model-based AJBR was 0.9 (95% CI, 0.7-1.2), and 0.4 (95% CI, 0.3-0.6) for major joint bleeds treated ≥2 days. The highest mean AJBR is found among participants aged 45 to 60 and ≥60 years. In a sensitivity analysis restricted to 214 participants without current inhibitors, AJBRs were comparable with all 232 participants (Supplementary Table S2).

### 3.4 Zero-treated bleeds

**Figure 3:**
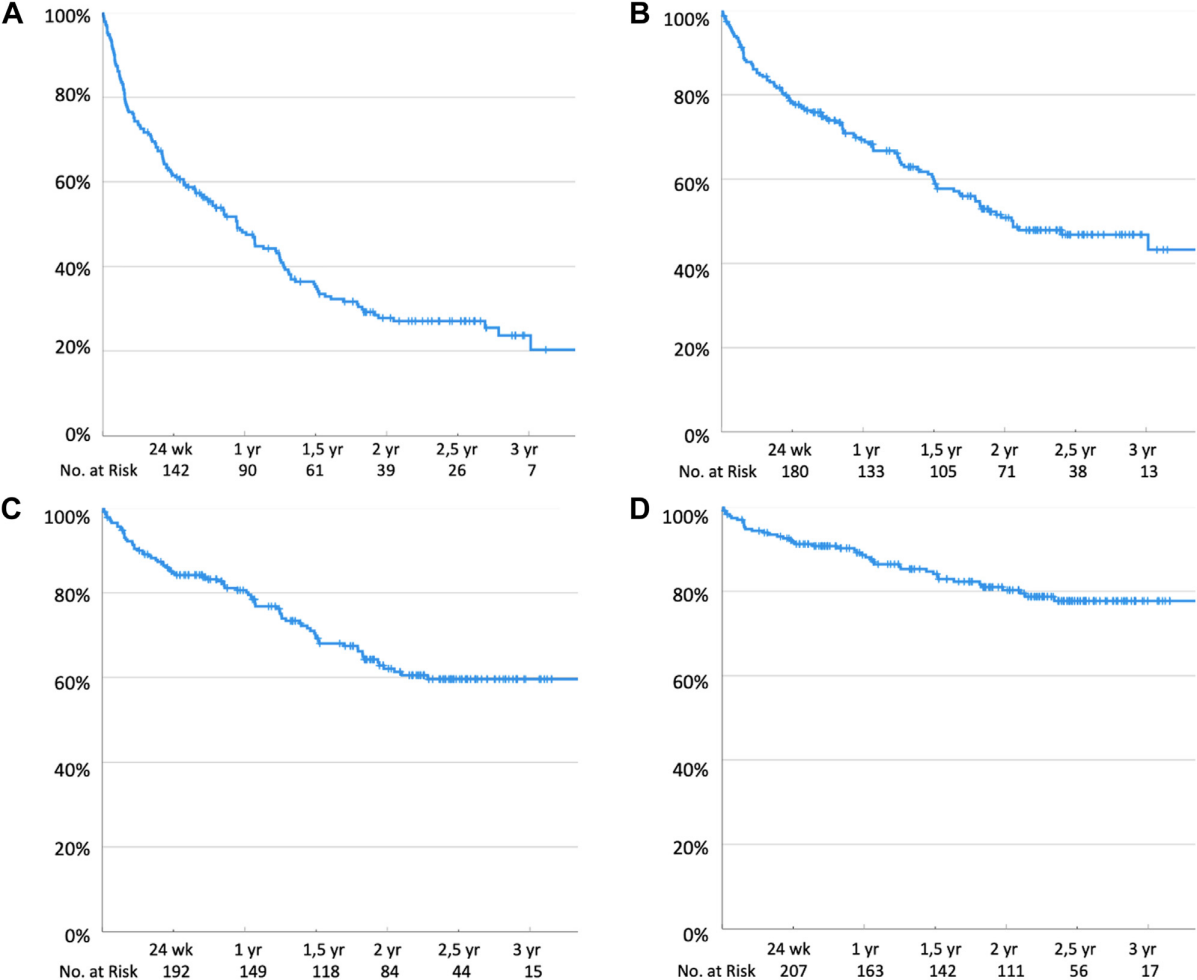
Kaplan–Meier survival curves for the proportion of participants with zero-treated (joint) bleeds. (A) Proportion of participants with zero-treated bleeds. At 24 weeks, 1 year, and 2 years, 63%, 48%, and 28% had zero treated bleeds, respectively. (B) Proportion of participants with zero-treated joint bleeds. At 24 weeks, 1 year, and 2 years, 80%, 69%, and 51% had zero-treated joint bleeds, respectively. (C) Proportion of participants with zero major joint bleeds treated with factor concentrate ≥2 days. At 24 weeks, 1 year, and 2 years, 86%, 81%, and 62% had zero joint bleeds treated with factor ≥2 days respectively. (D) Proportion of participants with zero major nontraumatic joint bleeds treated with factor concentrate ≥2 days. At 24 weeks, 1 year, and 2 years, 93%, 89%, and 80% had zero nontraumatic joint bleeds treated with factor ≥2 days respectively.

**Supplementary Figure S1:**
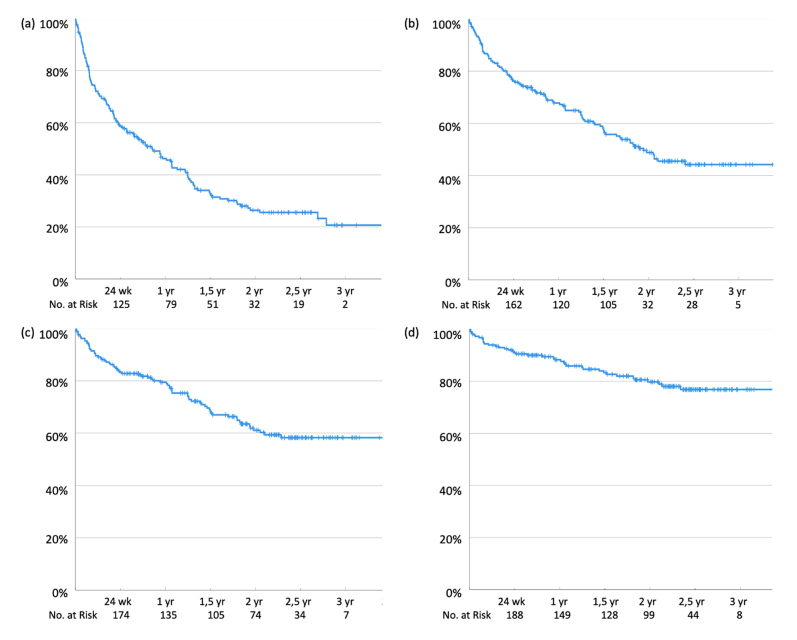


Figure 3 shows the survival analysis of zero-treated (joint) bleeds in the total study population. Supplementary Figure S1 shows these analyses for 214 participants without inhibitors.

Of the participants, 224 (97%) completed 24 weeks of follow-up. At 24 weeks, 63% of participants had zero-treated bleeds, 80% had zero-treated joint bleeds, and 90% had zero-treated nontraumatic joint bleeds. For major bleeds treated ≥2 days, 78% had zero major bleeds, 86% had zero major joint bleeds, and 93% had zero nontraumatic major joint bleeds. Zero bleed rates at 1 year and 2 years are presented in Figure 3. Supplementary Figure S1 shows the survival curves for all 214 participants without inhibitors. No clinically relevant differences can be observed.

### 3.5 Completeness of digital treatment diary data

Of 460 total identified bleeds, 255 (55%) were treated with standard half-life products and 166 (36%) with extended half-life products. The median (IQR) number of days a bleed was treated was 2 (1-3) days.

Of 460 bleeds, 44% (201/460) were documented in both digital treatment diaries and electronic health records, 24% (109/460) in digital treatment diaries only, and 32% (150/460) in electronic health records only, as presented in Table 3. Of all treated bleeds, 67% (310/460) were documented in digital treatment diaries. Compared with bleeds that were exclusively reported in electronic health records, diary-reported bleeds included a slightly higher proportion of joint bleeds (60% [65/109] vs 50% [75/150]) and were generally treated less extensively (26% [28/109] was treated ≥2 days vs 62% [93/150], respectively). The location of treatment (in the hospital or at home) could not always be determined due to inconsistent documentation in electronic health records.

**Table 3:** Registration of treated bleeds in digital patient diary.

Bleeds	Treated bleeds (*n* = 460)	Treated joint bleeds (*n* = 242)
Bleed is reported in:		
Treatment diary and electronic health record, *n* (%)	201 (44%)	102 (42%)
Treatment diary only, *n* (%)	109 (24%)	65 (27%)
Electronic health record only, *n* (%)	150 (32%)	75 (31%)
Proportion of bleeds that were recorded in the digital treatment diary	67% (310/460)	69% (167/242)
Proportion of bleeds that were recorded in the electronic health record	76% (351/460)	73% (177/242)

**Participants with ≥1 treated (joint) bleed**	**Participants with ≥1 treated bleed (*n* = 156)**	**Participants with ≥1 treated joint bleed (*n* = 107)**
Proportion of participants who did not report any bleed in their diary	56% (88/156)	49% (52/107)

**Participants with ≥2 treated (joint) bleeds**	**Participants with ≥2 treated bleeds (*n* = 103)**	**Participants with ≥2 treated joint bleeds (*n* = 53)**
Proportion of participants who did not report ≥50% of bleeds in their diary	41% (42/103)	40% (21/53)
Proportion of participants who did not report ≥75% of bleeds in their diary	18% (19/103)	21% (11/53)

Of 156 participants who experienced at least one treated bleed, 56% (88/156) did not document one or more treated bleeds in their digital treatment diary. Within these participants, a subgroup of 25% (39/156, including 17 children) was responsible for the majority of bleeds 67% (100/150) not documented in diaries. The other characteristics of patients in this subgroup were similar to the characteristics of all study participants.

## 4 Discussion

In this population-wide cohort study in the Netherlands, we assessed the real-world bleeding rates among people with severe hemophilia A using emicizumab, regardless of inhibitor status. We found mean treated ABR of 1.5 (95% CI, 1.3-1.7) and AJBR 0.8 (95% CI, 0.6-1.0). The proportions of participants with zero-treated bleeds and zero-treated joint bleeds were 63% and 80% at 24 weeks, respectively. Of all identified treated bleeds, 67% were reported in digital treatment diaries. For treated joint bleeds, this was 69%.

In our study, the negative-binomial regression model-based real-world (joint) bleeding rates were within the range of (joint) bleeding rates reported in the HAVEN studies summarized in Supplementary Table S3 [[3], [4], [5],[12], [13], [14]]. In the HAVEN studies that predominantly assessed participants without inhibitors, the mean model-based ABRs among people aged ≥12 years ranged from 1.0 (95% CI, 0.5-1.9) in HAVEN5 [14] to 2.4 (95% CI, 1.4-4.3) in HAVEN4 [12]. The mean model-based treated AJBRs in these studies ranged from 0.6 (95% CI, 0.3-1.2) in HAVEN5 [14] to 1.0 (95% CI, 0.3-3.3) in HAVEN4 [12].

Our bleeding rates are also comparable to those reported in other real-world studies, as presented in Supplementary Table S4 [6,[15], [16], [17], [18], [19], [20], [21], [22], [23], [24], [25], [26], [27], [28], [29], [30], [31], [32]]. We did not report median (IQR) bleeding rates, since those do not account for different follow-up times. Of 19 real-world studies, 11 assessed treated bleeds only, as we did [6,15,16,19,21,[24], [25], [26],[28], [29], [30]]. Of 11 studies that assessed treated bleeds only, the included number of participants ranged from 13 to 177, with heterogeneous populations of children and/or adults, participants with and/or without inhibitors, and participants with severe, moderate, and/or mild hemophilia. This hampers comparisons between studies. The median follow-up duration of these studies was shorter, with a median of 15.5 months among studies. The only study with a considerably longer median follow-up was a prospective cohort study among UK children and adults (39 months) [28]. Of 11 studies, 4 calculated negative-binomial regression-based bleeding rates [6,[28], [29], [30]]. In these 4 studies, the mean ABRs ranged between 0.2 and 1.1, and the mean AJBRs ranged between 0.1 and 0.4. Compared to our study, these A(J)BRs were slightly lower, possibly due to different study populations and the exclusive use of either electronic health record data or digital treatment dairy data. None of the real-world studies used both data sources. This might have resulted in an underestimation of bleeds. We assume that combining the 2 data sources better approaches the total number of bleeds.

In our study, the highest mean ABRs were found among adolescents aged 10 to 17 years and participants aged ≥45 years. The HAVEN studies did not further analyze different age groups. One other real-world study compared model-based bleeding rates among adolescents to the rest of the population. This prospective cohort study among UK children and adults with severe or moderate hemophilia A with an inhibitor also found that adolescents aged 12 to 18 years had the highest mean ABR (0.5 vs 1.9 in our study) [28].

Zero-treated bleed rates after the first 24 weeks of follow-up were comparable to the HAVEN studies [[3], [4], [5],[12], [13], [14]]. After 24 weeks, no comparison is possible due to different calculation methods used by Callaghan et al. [13]. Two real-world studies did report comparable zero bleed rates for 1 year [26,28].

### 4.1 Practical implications for secondary use of data

Over half of the participants did not document any bleeds in their treatment diary. Four potential reasons for incomplete documentation are as follows: individuals’ limited motivation to document bleeds, bleeds that were forgotten to be registered, hospital-treated bleeds that were already recorded in electronic health records, and technical problems with the digital treatment diary. Especially the first 2 reasons may expose the underlying cause for incomplete patient-reported data in health care in general. If individuals are not intrinsically motivated to report data and do not experience the beneficial effects of incentives with respect to relevant health outcomes or shared decision-making, this may cause incomplete registration due to lack of motivation. In the past, people using clotting factor concentrate prophylaxis had a stronger incentive to register bleeds (eg, treatment regimens were optimized by increasing doses when an increased number of bleeds was reported). In patients using emicizumab, the incentive may be unclear to patients as emicizumab doses are fixed. Moreover, since fewer bleeds occur on emicizumab, registration of bleeds might be more of a burden since the routine of registration is lost. Especially younger individuals and their parents may gradually become less accustomed to intravenous administrations at home and increasingly turn to hemophilia treatment centers for this. As a consequence, due to the lack of uniform guidelines on who documents hospital-treated bleeds, this could result in more missing bleed data.

To improve the completeness and quality of patient-reported data, we formulate 4 recommendations. First, to increase individuals’ motivation, we would recommend reducing the administrative burden for patients, by increasing the ease of use of digital tools and limiting the information that needs to be registered. Technical problems should be minimized. Second, to increase individuals’ motivation and perceived impact, individuals should receive feedback on reported bleed. This requires health care providers to review bleeds reported in digital treatment diaries during each consultation and emphasize that clinically relevant details of bleeding events are often forgotten if not immediately reported. Health care providers may especially focus on individuals who repeatedly did not report bleeds. Third, we recommend establishing clear guidelines for recording bleeds, including who documents hospital-treated bleeds. In the Netherlands, we recently issued instructions for both patients and health care providers. Finally, improved interoperability (ie, connectivity of different data sources) is critical to increasing health care providers’ motivation to consult digital treatment diaries. Especially in the long-term evaluation of therapies, patient-reported data should be accessible through (and automatically transferred to) electronic health records. The first step to overcome the technical challenges is to reach (nationwide) consensus on a (standardized) minimal data set that needs to be reported by individuals and health care providers, among others regarding bleeds. In the Netherlands, we recently achieved consensus on this data set [33].

### 4.2 Strengths and limitations

Strengths of this study were the nationwide real-world cohort, the use of multiple available data sources to best estimate bleeding rates, and the relatively long follow-up duration. By using negative-binomial regression, we were able to account for differences in follow-up durations.

Yet, several limitations should be considered. Our study population was younger than the overall Dutch hemophilia population [34]. Next, bleeding rates among nonusers of the digital treatment diary could not be determined. Although baseline characteristics of users and nonusers do not differ considerably, we were unable to compare bleeding rates. By only including participants who use the treatment diary, we might have selected a population that has higher health literacy. We did not compare health literacy between the 232 included participants and 124 nonusers of the digital treatment diary. Since health literacy is positively associated with treatment adherence, this could have resulted in the inclusion of a more adherent population. Since better adherence is associated with better control of bleeds, this might have resulted in an underestimation of bleeding rates in this study [35].

Furthermore, we analyzed self-reported bleeds that were not always confirmed by clinicians, so episodes of pain and swelling may have been misclassified as bleeds. However, all were treated with clotting factor concentrate. Moreover, self-reported bleeding rates are the golden standard in hemophilia research, so not all self-reported bleeds are confirmed by physical examination or imaging [[3], [4], [5],[12], [13], [14]]. Similar to our study, participants in the HAVEN trials reported bleeding events using a phone or tablet and contacted health care providers at their own discretion [[3], [4], [5],[12], [13], [14]]. Therefore, bleeding rates may be an overestimation, since aches and pains may have been misinterpreted as (joint) bleeds. The assessment of (joint) bleeds treated with factor concentrate ≥2 days in our study represents an analysis of a subset of major bleeds [13].

Additionally, misclassifications of the traumatic vs nontraumatic cause of bleeds could have occurred. Due to inconsistent documentation in electronic health records, the treatment location could not always be determined. Moreover, we did not check for adherence to emicizumab prophylaxis, although this unlikely had a large effect on our findings due to the long half-life of emicizumab.

Bleed registration in the digital diary may have been suboptimal because users and treaters were not specifically instructed to record all bleeds. It might have been unclear which party would record bleeds treated at the hemophilia treatment center or which were already discussed by telephone or teleconsulting with the hemophilia treatment center. Additionally, since participants spontaneously self-reported side effects in their digital treatment diary, reported side effects likely represent an underestimation of (mild) side effects. Finally, there were occasional technical problems with the treatment diary app.

### 4.3 Future research

Future real-world research could evaluate the effects of the 4 interventions intended to improve the adoption and use of digital treatment diaries among individuals and health care providers. By re-evaluating the discrepancies between digital treatment diaries and electronic health records, and comparing discrepancies to this study, we will be able to evaluate the efficacy of current interventions and identify where further improvements are needed. Besides combining electronic health records and digital treatment diary data, including medication dispense data could further help to better capture all bleeds. Finally, since there is large heterogeneity in reporting real-world bleeding rates, there is a strong need for developing reporting standards to better compare across studies and accumulate data. These standards should address: the reporting of results and follow-up in defined subgroups (based on age classes and hemophilia severity), the definition of a bleeding episode (eg, treated bleeds), the definitions of traumatic and spontaneous bleed, and the method of estimating bleeding rates (eg, annualized rate based on a binomial model).

### 4.4 Conclusion

Real-world annual treated bleeding rates among Dutch people with severe hemophilia A using emicizumab calculated using treatment diary and electronic health record data were comparable to other real-world studies. By exclusively using digital treatment diary data, 33% of (joint) bleeds could be missed. These discrepancies underline the need for better guidelines on the use of the treatment diary to improve data quality and the importance of discussing patient-reported data in consultations to promote adherence to digital patient diaries.

## Supplementary material

Supplementary Appendices
