# Supplementary material for: Real-world bleeding rates on emicizumab: the value of using nationwide digital treatment diary data in clinical research
Source: Res Pract Thromb Haemost. 2025 Feb 27;9(2):102717. doi: 10.1016/j.rpth.2025.102717 (PMC11981740; doi:10.1016/j.rpth.2025.102717)
Supplement: Supplementary Appendices [file mmc1.docx]

**Supplemental table S1: Comparison of 232 included participants on emicizumab prophylaxis who use the digital treatment diary vs. 124 participants on emicizumab prophylaxis who do not use the digital treatment diary**

|  | Digital treatment diary-users,  included in the patient registry (n=232) | | | People not using the digital treatment diary,  included in the patient registry (n=124) | | |
| --- | --- | --- | --- | --- | --- | --- |
|  | All participants  (n=232) | Children  (n=85) | Adults  (n=147) | All  (n=124) | Children  (n=58) | Adults  (n=66) |
| Age in years at the start of emicizumab, median (IQR) | 27 (13-51) | 8 (3.5-14.5) | 44 (29-59) | 19 (8-42) | 7.5 (3-13) | 40 (27-59) |
| On demand treatment at the start of emicizumab, n (%) | 11 (5%) | 11 (15%) | 0 | 9 (7%) | 5 (9%) | 4 (6%) |
| HIV infection, n (%) | 11 (5%) | 0 | 11 (8%) | 0 | 0 | 0 |
| Hepatitis C infection, n (%)  Current infection  Treated or cleared  Never infected  Missing | 2 (1%)  71 (30%)  159 (69%)  0 | 0  0  85 (100%)  0 | 2 (2%)  71 (48%)  74 (50%)  0 | 4 (3%)  29 (23%)  85 (69%)  6 (5%) | 0  0  58 (100%)  0 | 4 (6%)  29 (44%)  27 (41%)  6 (9%) |
| Inhibitor, n (%)  Current inhibitor  Past inhibitor  Never had an inhibitor  Missing | 18 (8%)  37 (16%)  177 (76%)  0 | 11 (14%)  12 (14%)  62 (72%)  0 | 7 (5%)  25 (17%)  115 (78%)  0 | 7 (6%)  28 (23%)  80 (64%)  9 (7%) | 5 (9%)  13 (22%)  35 (60%)  5 (9%) | 2 (3%)  15 (23%)  45 (68%)  4 (6%) |
| Emicizumab prescription (initial)^#^, median (IQR)  Injection frequency, once every n week(s)  Missing  Dose in mg/kg/week  Missing | 2 (1-2)  0  1.48 (1.40-1.57)  52 (22%) | 2 (2-3)  0  1.50 (1.34-1.65)  41 (48%) | 2 (1-2)  0  1.48 (1.40-1.56)  11 (7.5%) | 1.5 (1-2)  24 (19%)  1.50 (1.40-1.61)  46 (37%) | 2 (2-3)  10 (17%)  1.48 (1.30-1.64)  30 (63%) | 1 (1-1)  14 (21%)  1.50 (1.43-1.58)  16 (24%) |
| Follow-up duration in months^*^, median (IQR)  Missing | 27 (14-31)  0 | 29 (22-32)  0 | 25 (11-31)  0 | 24 (16-35)  1 (1%) | 31 (22-36)  0 | 22 (14-31)  1 (2%) |

IQR: interquartile range

# Dose and frequency of emicizumab prophylaxis after the loading dose phase of 28 days.

*The follow-up period starts 28 days after the start of emicizumab prophylaxis, until the moment of data collection (October 1^st^ 2023) or the stop date of emicizumab prophylaxis.

**Supplemental table S2: Model-based bleeding rates for 214 participants without inhibitors on emicizumab, per age category**

|  | All |  | Children | | |  | Adults | | | |
| --- | --- | --- | --- | --- | --- | --- | --- | --- | --- | --- |
|  | participants  (n=214) |  | All children  (n=74) | 0-9yr  (n=36) | 10-17yr  (n=38) |  | All adults  (n=140) | 18-30yr  (n=45) | 31-60yr  (n=65) | >60yr  (n=30) |
| ABR, mean (95% CI)  Treated bleeds  Bleeds treated ≥2 days | 1.6 (1.4-1.9)  0.8 (0.7-1.0) |  | 1.5 (1.2-1.9)  0.7 (0.5-1.0) | 1.1 (0.8-1.6)  0.3 (0.2-0.5) | 1.9 (1.3-2.6)  1.2 (0.8-1.6) |  | 1.6 (1.4-2.0)  0.8 (0.7-1.0) | 1.2 (0.9-1.7)  0.7 (0.5-1.0) | 1.7 (1.3-2.3)  0.9 (0.7-1.1) | 2.2 (1.5-3.4)  1.1 (0.6-1.8) |
| AJBR, mean (95% CI)  Treated joint bleeds  Joint bleeds treated ≥2 days | 0.9 (0.7-1.1)  0.4 (0.3-0.6) |  | 0.7 (0.5-0.9)  0.4 (0.3-0.5) | 0.5 (0.3-0.8)  0.2 (0.1-0.4) | 0.8 (0.6-1.2)  0.6 (0.4-0.8) |  | 1.0 (0.7-1.3)  0.5 (0.3-0.6) | 0.5 (0.3-0.8)  0.3 (0.1-0.4) | 1.1 (0.8-1.6)  0.5 (0.3-0.9) | 1.3 (0.7-2.3)  0.6 (0.3-1.2) |
| Traumatic AJBR, mean (95% CI)  Treated joint bleeds  Joint bleeds treated ≥2 days | 0.4 (0.3-0.5)  0.2 (0.1-0.2) |  | 0.5 (0.3-0.7)  0.2 (0.2-0.4) | 0.4 (0.3-0.8)  0.2 (0.1-0.3) | 0.4 (0.3-0.7)  0.3 (0.2-0.5) |  | 0.3 (0.2-0.5)  0.1 (0.1-0.2) | 0.3 (0.1-0.5)  0.1 (0.1-0.3) | 0.4 (0.2-0.6)  0.2 (0.1-0.3) | 0.3 (0.2-0.6)  0.2 (0.1-0.4) |
| Non-traumatic AJBR, mean (95% CI)  Treated joint bleeds  Joint bleeds treated ≥2 days | 0.5 (0.3-0.6)  0.3 (0.2-0.4) |  | 0.2 (0.1-0.3)  0.2 (0.1-0.3) | 0.0 (0.0-0.2)  0.0 (0.0-0.1) | 0.4 (0.2-0.6)  0.3 (0.2-0.5) |  | 0.6 (0.4-0.9)  0.3 (0.2-0.5) | 0.2 (0.1-0.4)  0.1 (0.0-0.3) | 0.7 (0.4-1.1)  0.4 (0.2-0.7) | 0.9 (0.4-1.9)  0.5 (0.2-1.1) |

ABR; annual bleeding rate, AJBR; annual joint bleeding rate, CI; confidence interval, yr; years

**Figure S1: Kaplan-Meier survival curves for the proportion of 214 participants without inhibitors with zero treated (joint) bleeds**


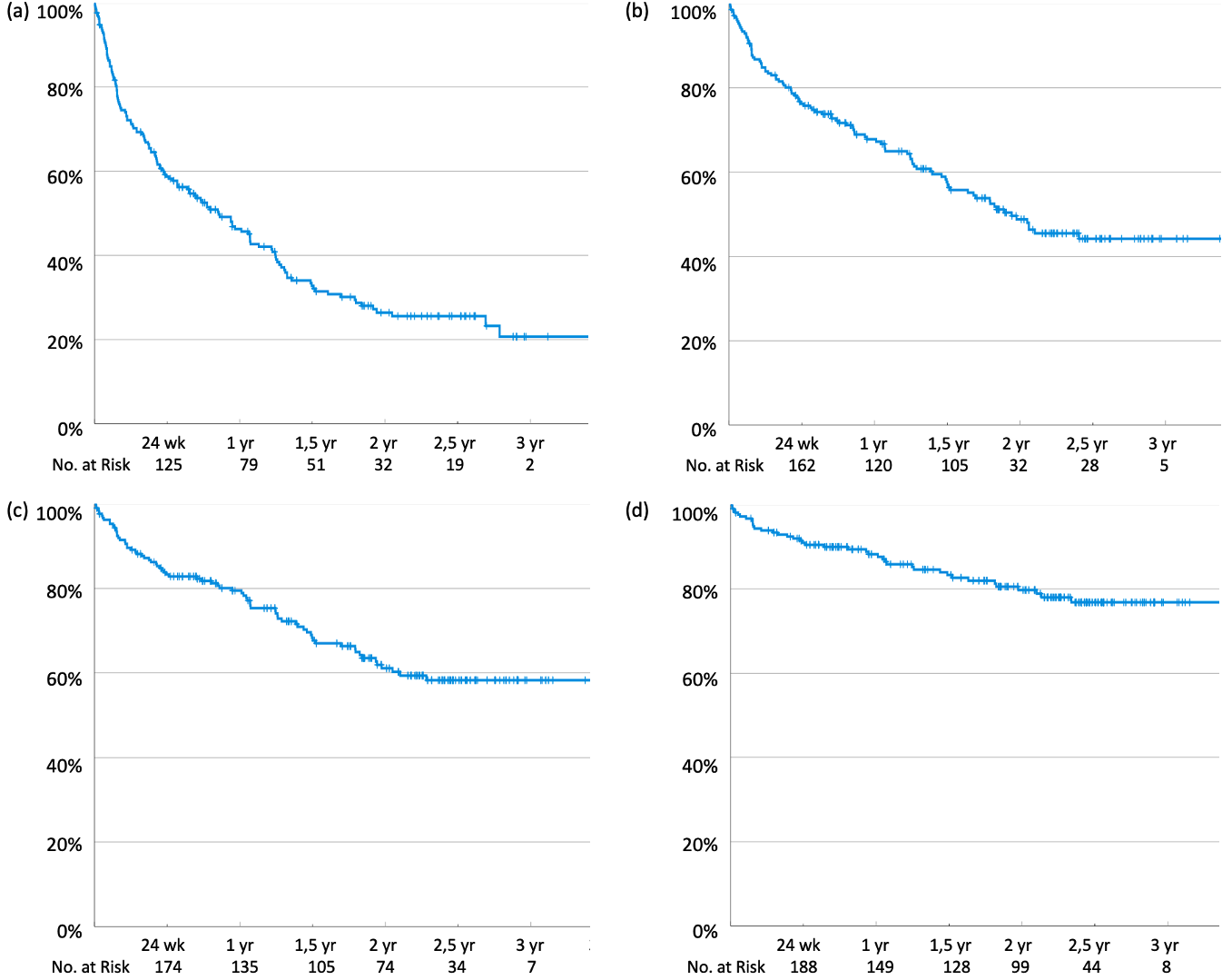


Figure S1a: Proportion of participants with zero treated bleeds. At 24 weeks, 1 year and 2 years, Figure S1b: Proportion of participants with zero treated joint bleed. At 24 weeks, 1 year and 2 years,

61%, 46%, and 26% had zero treated bleeds respectively. 78%, 68% and 49% had zero treated joint bleeds respectively.

Figure S1c: Proportion of participants with zero major joint bleeds treated with factor Figure S1d: Proportion of participants with zero major non-traumatic joint bleeds treated with concentrate ≥2 days. At 24 weeks, 1 year and 2 years, 85%, 80%, and 61% had factor concentrate ≥2 days. At 24 weeks, 1 year and 2 years, 92%, 88% and 80% had

zero joint bleeds treated with factor ≥2 days respectively. zero non-traumatic joint bleeds treated with factor ≥2 days respectively.

**Supplemental Table S3: Summary of HAVEN-trials including participants with severe haemophilia A**

|  | n | Age (in years) | Inhibitor* | Mean (95% CI)  model-based ABR | Mean (95% CI)  model-based AJBR | Zero treated bleeds  (24 weeks) | Zero treated bleeds  (1 year) |
| --- | --- | --- | --- | --- | --- | --- | --- |
| HAVEN1 | 109 | ≥12 | + | 2.9 (1.7-5.0) | 0.8 (0.3-2.2) | All: 63% | NR |
| HAVEN2 | 88 | <12 | + | 0.3 (0.2-0.5) | 0.2 (0.1-0.3) | NR | All: 77%  Joint: 85% |
| HAVEN3 | 152 | ≥12 | - | 1.3 (0.9-2.3) | 0.9 (0.4-1.7) | All: 60%  Joint: 74% | NR |
| HAVEN4 | 41 | ≥12 | +I: 5%  -I: 95% | 2.4 (1.4-4.3) | 1.0 (0.3-3.3) | All: 56%  Joint: 71% | NR |
| HAVEN1-4 | 401 | All | +I: 52%  -I: 48% | 1.4 (1.1-1.7) | 0.9 (0.7-1.2) | All: 82% ^**^  Joint: 90% ^**^ | NR |
| HAVEN5 | 70 | ≥12 | +I: 23%  -I: 77% | 1.0 (0.5-1.9) | 0.6 (0.3-1.2) | All: 78%  Joint: 69% | NR |

Abbreviations: +I: inhibitor present at study entry; -I: no inhibitor present at study entry; CI, confidence interval; IQR, interquartile range; n, number of included participants; NR, not reported.

All annualized bleeding rates reported in this table are bleeding rates for treated bleeds only. HAVEN 6 and 7 are not included in this table, due to their lesser relevance to our study. HAVEN6 included persons with moderate and mild haemophilia. HAVEN7 included only children aged <1 year.

* Whether or not persons with presence of a current inhibitor were included.

** Proportions describe the final 24 weeks of a participants of the study period.

**Supplemental Table S4 : Summary of all real-world studies published before September 1^st^, 2024**

|  |  |  |  |  |  | Follow-up (in months) | | ABR** | | ABR | | AJBR** | | AJBR | | Zero bleeds | |
| --- | --- | --- | --- | --- | --- | --- | --- | --- | --- | --- | --- | --- | --- | --- | --- | --- | --- |
|  | **Design** | **n** | **Participants** | **Severity** | **Inhi-bitor*** | **Median** | **Range and/or IQR** | **Mean** | **SD or 95%CI** | **Median** | **Range or IQR** | **Mean** | **SD or 95%CI** | **Median AJBR** | **Range or IQR** | **Cut off** | **No cut off***** |
| 11 studies including treated bleeds only (n=893 participants) | | | | | | | | | | | | | | | | | |
| Barg ‘20 | Prosp. cohort | 40 | Israeli children | Severe | + and - | 10 | Range 2-24 | NR | NR | 1 | IQR 0-3 | NR | NR | NR | NR | NR | 49% |
| Barg ‘21 | Prosp. cohort | 107 | Israeli children, adults | Severe | + and - | Ch: 18  Ad: 13 | Ch: IQR 9-21  Ad: IQR 8-21 | NR | NR | Ch: 0.25 Ad: 0.5 | Ch: IQR 0-2  Ad: IQR 0-2 | NR | NR | NR | NR | NR | Ch: 50%  Ad: 49% |
| Donners ‘22 | Retro. cohort | 112 | Dutch children, adults | Severe, moderate, mild | + and - | 13 | IQR 7-19  Range 0-48 | Ch: 0.7^##^  Ad: 0.8^##^ | Ch: 0.4-1.2  Ad: 0.6-1.2 | NR | NR | Ch: 0.2^##^  Ad: 0.4^##^ | Ch: 0.1-0.5  Ad: 0.3-0.7 | NR | NR | NR | NR |
| Glonnegger ‘22 | Retro. cohort | 13 | German children | Severe, moderate | + and - | 24 | Range 1-40 | NR | NR | 0 | Range 0-0.5 | NR | NR | NR | NR | NR | NR |
| Hassan ‘22 | Retro. cohort | 51 | UK children | Severe | + | 15 | Range 3-39  IQR 10-18 | NR | NR | NR | NR | NR | NR | NR | NR | NR | 80% |
| McCary ‘20 | Retro. cohort | 93 | US children, adults | Severe, moderate | + and - | +I: 14  -I: 8 | NR | +I: 0.4  -I: 0.4 | +I: 0-0.9  -I: 0.2-0.6 | 0 | NR | +I: 0.4  -I: 0.1 | +I: 0-1.2  -I: 0-0.2 | NR | NR | +I: 95% at 6 months  -I: 89% at 6 months | NR |
| Misgav ‘20 | Prosp. cohort | 17 | Israeli elderly | Severe | + and - | 13 | Range 3-26  IQR 7-16 | NR | NR | +I: 0.5  -I: 1.0 | +I: Range 0-3  -I: Range 0-10 | NR | NR | NR | NR | NR | 29% |
| Shen ‘23 | Retro. cohort | 39 | Taiwanese children, adults | Severe | + | NR | NR | 2 | NR | 0 | NR | 1 | NR | 0 | NR | Y1 51%^#^  Y2 56%^#^  Y3 81%^#^ | NR |
| van der Zwet, ‘24 | Prosp. cohort | 177 | Children | Severe, moderate, mild | + and - | 16 | IQR 12-25 | +I: 0.8^##^  -I: 1.1^##^ | NR | NR | NR | +I: 0.3^##^  -I: 0.3^##^ | NR | NR | NR | NR | NR |
| Wall ‘22 | Prosp. cohort | 117 | UK children, adults | Severe, moderate | + | 39 | IQR 29-45 | 0.3^##^ | 0.2-0.6 | 0 | IQR 0-0 | 0.2^##^ | 0.1-0.4 | 0 | IQR 0-0 | 1 year: 89% | 88% |
| Xu ‘24 | Retro. cohort | 127 | Chinese children, adults | Severe, moderate | + and - | 16 | IQR 9-30 | 0.2^##^ | 0.1-0.3 | 0 | IQR 0-0 | 0.1^##^ | 0-0.1 | 0 | IQR 0-0 | NR | 80% |
| 8 studies including treated and non-treated bleeds (n=444 participants) | | | | | | | | | | | | | | | | | |
| Boulden Warren ‘20 | Retro. cohort | 68 | US children, adults | Severe | + and - | 7 | Range 6-41 | NR | NR | 2.3 | IQR 0-2.5 | NR | NR | NR | NR | NR | 37% |
| Ebbert ‘19 | Retro. cohort | 43 | US children, adults | Severe, moderate, mild | + and - | +I: 12  -I: 5 | +I: Range 1-16  -I: Range 0-12 | +I: 0.5  -I: 0.9 | +I: 0.1  -I: 0.4 | NR | NR | +I: 0.2  -I: 0.1 | +I: 0.1  -I: 0.1 | NR | NR | NR | 66% |
| Escobar ‘23 | Retro. cohort | 131 | US children, adults | NR | - | 18 | Range 7-29  IQR 12-22 | 0.2 | 0.7 | NR | NR | 0.2 (Sp.) | 0.7 | NR | NR | NR | NR |
| Hahn ‘23 | Retro. cohort | 16 | Korean children, adults | NR | + | 24 | Range 12-59 | 0.35 | 0.5 | 0 | IQR 0-1.5 | 0.1 (SD 0.3) | 0.3 | 0 | IQR 0-1.2 | NR | 56% |
| Levy-Mendelovich ‘21 | Prosp. cohort | 70 | Israeli children, adults | Severe | + and - | >18 | NR | NR | NR | NR | NR | NR | NR | NR | NR | NR | Tr. 39%  Sp.29% |
| Liu ‘22 | Prosp. cohort | 13 | Chinese children | Severe, moderate | + and - | 18 | Range 6-26 | NR | NR | 0.5 | Range 0-4 | NR | NR | 0 | Range 0-1.1 | NR | 46% |
| Sun ‘22 | Retro. cohort | 85 | US children, adults | Severe, moderate | - | >6 | NR | 0.6 | 0.4-0.9 | NR | NR | 0.4 | 0.2-0.5 | NR | NR | NR | NR |
| Wei Lee ‘23 | Retro. cohort | 18 | Singaporean children, adults | Severe, moderate, mild | + and - | 22 | Range: 3-46 | NR | NR | 0 | IQR 0-0 | NR | NR | NR | NR | 83% at 6 months | NR |

Abbreviations: +I, with inhibitor; -I, without inhibitor, Ad., adult; Ch., child; CI, confidence interval; IQR, interquartile range; n, number of included participants, Sp., spontaneous, Tr. traumatic

* Whether or not the presence of a current inhibitor was or was not an inclusion criteria.

** All mean A(J)BRs are not negative-binomial regression model-based, unless specifically indicated with ##.

*** The proportions reported in this column were proportion of zero treated (joint) bleeds that were *not* determined at fixed follow-up periods (e.g. at 24 weeks), but at the end of follow-up (which is different per participant). This hinders their interpretation.

# The proportion of participants were zero bleeds were found per year. For example, in the third year of follow-up, the study by Shen found that 81% of participants reported zero treated bleeds.

## Model-based calculation of the mean ABR or AJBR.
